# Comparison of Canine and Feline Meningiomas Using the Apparent Diffusion Coefficient and Fractional Anisotropy

**DOI:** 10.3389/fvets.2020.614026

**Published:** 2021-01-11

**Authors:** Masae Wada, Daisuke Hasegawa, Yuji Hamamoto, Yoshihiko Yu, Rikako Asada, Aki Fujiwara-Igarashi, Michio Fujita

**Affiliations:** ^1^Laboratory of Veterinary Radiology, Nippon Veterinary and Life Science University, Musashino, Japan; ^2^ORM Co.Ltd., Saitama, Japan; ^3^The Research Center for Animal Life Science, Nippon Veterinary and Life Science University, Musashino, Japan

**Keywords:** cats, diffusion tensor imaging, diffusion-weighted imaging, dogs, magnetic resonance imaging, meningioma

## Abstract

Meningiomas are the most common intracranial tumor in dogs and cats, and their surgical resection is often performed because they are present on the brain surface. Typical meningiomas show comparatively characteristic magnetic resonance imaging findings that lead to clinical diagnosis; however, it is necessary to capture not only macroscopic changes but also microstructural changes to devise a strategy for surgical resection and/or quality of removal. To visualize such microstructural changes, diffusion-weighted imaging (DWI) and diffusion tensor imaging (DTI) have been used in human medicine. The aim of this retrospective study was to investigate the different characteristics of the apparent diffusion coefficient (ADC) from DWI and fractional anisotropy (FA) from DTI of meningioma between dogs and cats. Statistical analyses were performed to compare ADC and FA values between the intratumoral or peritumoral regions and normal-appearing white matter (NAWM) among 13 dogs (13 lesions, but 12 each in ADC and FA analysis) and six cats (seven lesions). The NAWM of cats had a significantly lower ADC and higher FA compared to dogs. Therefore, for a comparison between dogs and cats, we used ADC and FA ratios that were calculated by dividing the subject (intra- or peritumoral) ADC and FA values by those of NAWM on the contralateral side. Regarding the intratumoral region, feline meningiomas showed a significantly lower ADC ratio and higher FA ratio than canine meningiomas. This study suggested that ADC and FA may be able to distinguish a meningioma that is solid and easy to detach, like as typical feline meningiomas.

## Introduction

Meningiomas are the most common intracranial tumor in dogs and cats. The diagnostic rate of meningiomas has been increasing (1–4) due to technological advancements in diagnostic imaging. In general, meningioma is often a solitary mass broadly attached to the meninges and shows isointense or slightly hypo- to hyperintense signals on T1-weighted (T1W) images and variably hypointense to hyperintense signals on T2-weighted (T2W) images on magnetic resonance imaging (MRI) (4, 5). Marked and relatively homogeneous contrast enhancement with the dural tail sign is also typically observed (6). Due to some of these characteristic MRI findings, meningiomas can be presumptively diagnosed by conventional sequences, although the definitive diagnosis is only made with histopathology. As a treatment, surgical resection is often selected for canine and feline meningiomas (7, 8). Especially in feline meningiomas, surgical resection is associated with a significantly longer survival time after diagnosis than other treatment strategies (1, 2). The consistency of meningiomas is an important factor in developing a strategy for surgical resection and predicting the degree of removal; a solid tumor is easily peeled off, whereas a fragile tumor may be removed by suctioning. Diffusion-weighted imaging (DWI) and diffusion tensor imaging (DTI) have been used to predict the consistency of the tumors in human medicine (9, 10).

The apparent diffusion coefficient (ADC) and fractional anisotropy (FA), which are derived from DWI and DTI, respectively, provide information on water diffusion, i.e., Brownian movement of water molecules, in tissue. The ADC value correlates with tumor cellularity (11) and the amount of fibrous tissue (12). The FA value is statistically significant with regard to meningioma consistency, i.e., higher FA values are associated with hard tumors (10). In humans, hard meningiomas have lower ADC values (9) and higher FA values (10) than soft meningiomas. Most feline meningiomas tend to be firm, well-encapsulated, and easily delineated from the surrounding normal brain (8, 13). Therefore, the mass will be removed totally or occasionally divided into small portions using forceps and scissors. Conversely, canine meningiomas are often soft and fragile, and also affect to the adjacent brain tissue pathologically. Due to the poorly defined border in canine meningiomas, a complete surgical removal is sometimes difficult even used bipolar electrocautery and (ultrasonic) surgical aspirator (14, 15).

As feline meningiomas are often found to be firm when palpated intraoperatively, we hypothesized that there would be a significant difference when comparing ADC and FA values between canine and feline meningiomas, i.e., feline meningiomas will have lower ADC and higher FA than those of canine meningiomas. Besides, a strong mass effect, such as the condition where the surrounding tissues are compressed and displaced and a mass finally induces severe brain herniation, is more commonly observed in cats with meningioma than in dogs (16–18). For that reason, in the peritumoral region, we hypothesized that feline meningiomas would have lower ADC and higher FA values than canine meningiomas due to the presence of severely compressed brain tissue by the tumor. In order to verify these hypotheses, this retrospective study compared the ADC and FA values of normal-appearing white matter (NAWM) contralateral to the lesion between dogs and cats, and then compared intratumoral and peritumoral ADC and FA values between feline and canine meningiomas.

## Materials and Methods

This was a retrospective analytical study that was performed using the archived medical and imaging records of all dogs and cats with histologically confirmed intracranial meningioma that were referred to the Veterinary Medical Teaching Hospital of the Nippon Veterinary and Life Science University and that were subjected to DWI and/or DTI in addition to conventional MRI sequences between March 2013 and March 2016. All patients were presented to the teaching hospital with clinical signs suggesting a forebrain lesion, and all MRIs used in this study were performed before surgery, radiation therapy, or chemotherapy. All tumoral lesions were resected surgically as a primary treatment or were collected by necropsy after death, and diagnosed definitively as meningioma by histopathological evaluation. As the nature of this study was retrospective imaging analysis, ethical consent for animal use was not requested. Nevertheless, all owners of the dogs and cats included in this study had accepted to the use of their data for academic purposes, and had previously signed a consent form at the first presentation to the teaching hospital.

### MRI Techniques

All MRI studies were performed using a 3.0 Tesla superconducting system (Signa HDxt; GE Healthcare, Tokyo, Japan) with an eight-channel human knee coil as the RF coil. Routine MR sequences were included: 3D- or 2D-T2W, T2-fluid-attenuated inversion recovery (FLAIR), T1-FLAIR, and 3D-T1W or 2D-T1-FLAIR with and without contrast enhancement (CE-T1W). Acquisition parameters of these sequences are; 3D-T2W (3D-T2 Cube) images were obtained in sagittal plane with fast spin echo (FSE), with TR/TE = 3,200/78–90 (auto) ms, slice thickness (ST) = 0.6 mm, slice gap (SG) = 0 mm, FOV = 15 × 15 cm, matrix = 512 × 512, and number of acquisitions (NAQ) = 1. 3D-T1W images were obtained in sagittal plane with the spoiled gradient recalled acquisition in the steady state (SPGR) sequence, with TR/TE = 6.5/3.1 ms, ST = 0.6 mm, SG = 0 mm, FOV = 15 × 15 cm, matrix = 256 × 192, and NAQ =1. 2D-T2W (FSE; TR/TE = 7,000/82 ms), T2-FLAIR (SE; TR/TE/TI = 11,002/141.8/2,400 ms), and T1-FLAIR (FSE; TR/TE/TI = 2,994/8.4/920 ms) images were obtained in transvers plane at ST = 2.0 mm, SG = 0.5 mm, FOV = 15 × 15 cm, matrix = 512 × 512, and NAQ = 2, respectively. A contrast agent, Gadodiamide (Omniscan; Daiichi-Sankyo, Tokyo, Japan) was administered intravenously at 0.05–0.1 mmol/kg in contrast-enhanced sequences.

The DWI sequence was acquired in the transverse plane using a periodically rotated overlapping parallel lines with enhanced reconstruction (PROPELLER) sequence with the following parameters: TR/TE = 8,000/72.2 ms; diffusion gradient encoding in three orthogonal directions; b value = 1,000 s/mm^2^; FOV = 15 × 15 cm, matrix = 256 × 256; ST = 2.0 mm; SG = 0.5 mm; NAQ = 1; and total slice number = 20–32 (which varied depending on each case to cover the whole brain).

The DTI sequence was also acquired in the transverse plane using a single-shot echo-planar imaging sequence with the following parameters: TR/TE = 8,000/89.6 ms; diffusion gradient encoding in 15 directions; b value =1,000 s/mm^2^; FOV 15 × 15 cm; matrix = 256 × 256; ST = 2.4 mm; NAQ = 2; and total slice number = 20–32 (which varied depending on each case to cover the whole brain). The diffusion studies were performed before the intravenous administration of contrast agent.

### Measurements of Diffusion Parameters

**Figure 1:**
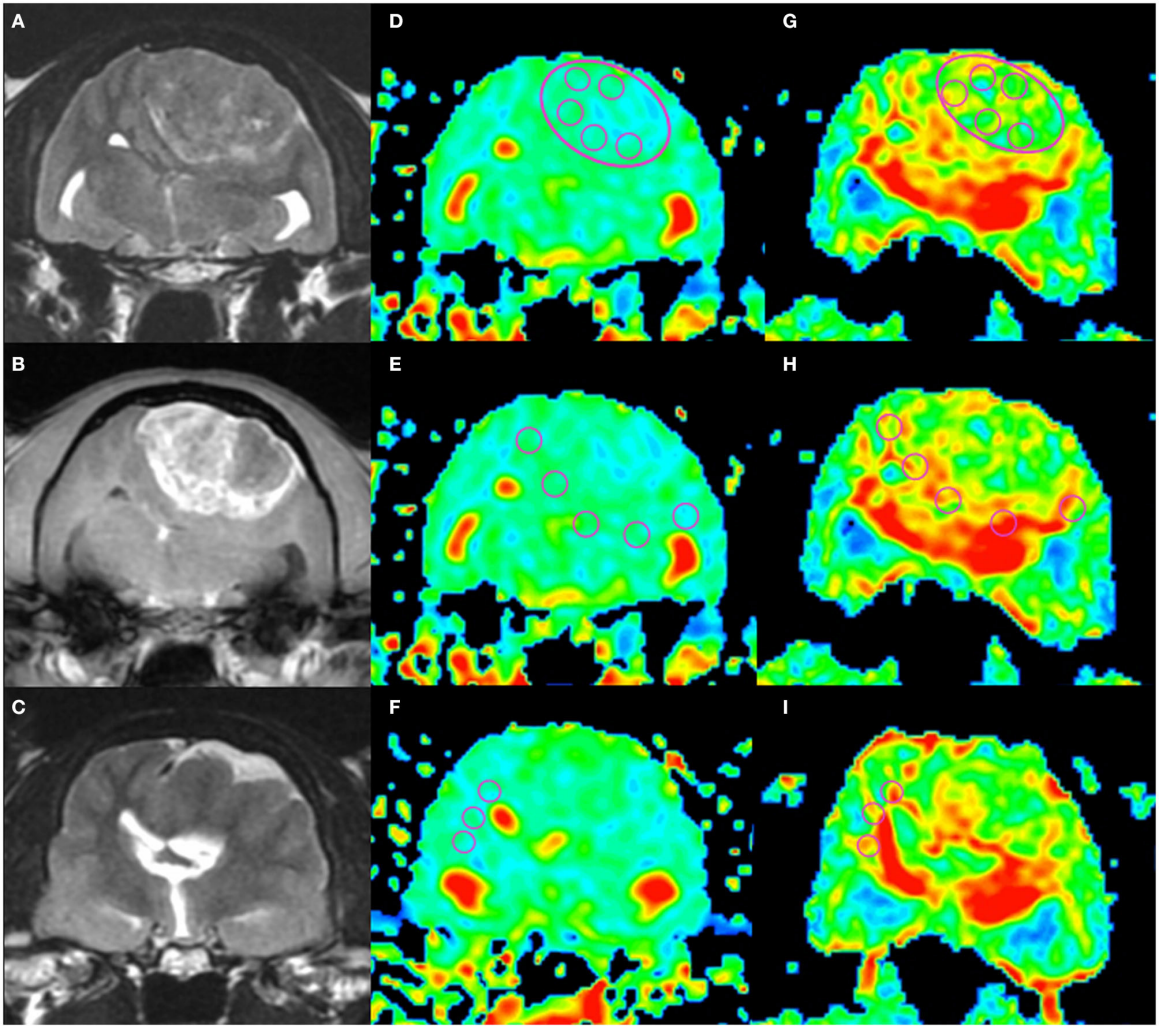
An example of apparent diffusion coefficient (ADC) and fractional anisotropy (FA) measurements in a cat (Cat 1). Localizing transverse T2-weighted imaging (T2WI) and T1-weighted imaging with contrast enhancement (CE-T1WI) with superimposed ADC and FA color maps. The upper row shows the intratumoral region (“revolver technique”), the middle row shows the peritumoral regions, and the lower row shows the contralateral normal-appearing white matter region. T2WI **(A)**, CE-T1WI **(B)** of the tumor area, and T2WI (rostral from A and B) of the normal-appearing white matter area **(C)**. Regions of interest (circles) were placed on the ADC maps **(D–F)** and FA maps **(G–I)** as in the figure.

**Figure 2:**
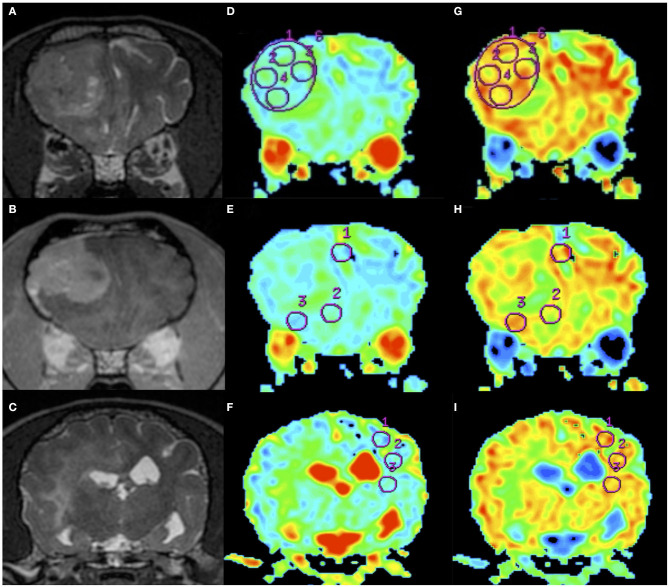
An example of apparent diffusion coefficient (ADC) and fractional anisotropy (FA) measurements in a dog (Dog 6). Order of images is same as Figure 1. T2WI **(A)**, CE-T1WI **(B)** of the tumor area, and T2WI (caudal from A and B) of the normal-appearing white matter area **(C)**. Regions of interest (circles) were placed on the ADC maps **(D–F)** and FA maps **(G–I)** as in the figure.

All diffusion parameters were measured by one operator (MW) in a non-blinded manner, and the operator could access all information for each case. ADC and FA maps were created by calculating the signal intensities on DWI and DTI at two b-values (0 and 1,000 s/mm^2^) with the internal software (Functool; GE Healthcare, Tokyo, Japan) of the MRI system. ADC and FA maps were transformed into a color code based on pixel-by-pixel ADC and FA values, respectively. As most tumors were isointense on diffusion images, the tumoral lesion could not be detected by visual assessment of the diffusion images alone. In the ADC and FA color maps, the boundary between the tumor and surrounding brain area was unclear; therefore, those maps were superimposed on the T2W or CE-T1W images for the placement of the ROIs. For each patient, five regions of interest (ROIs) were placed manually within each of the intratumoral, peritumoral, and contralateral NAWM. The method for arranging the ROIs was the same as that described previously (19). Concisely, for measuring the intratumoral region, a specific ROI method, referred to the “revolver technique,” was applied (20). A large circular ROI was placed to cover as much of the interior border of the tumor as possible, with an area ranging from 10.9 to 227.3 mm^2^ depending on tumor size. Five large ROIs were placed on the multiple slices or occasionally on the same slice (depending on slice number showing the tumor). In addition, five small circular ROIs with an area ranging from 7.7 to 17.2 mm^2^ were placed within the large ROI. Another five ROIs were placed along the peritumoral region, which was defined to be within 10 mm from the tumoral margin presenting with contrast enhancement in CE-T1W images (Figures 1, 2). As seen as Figures 1, 2, the peritumoral ROIs contained both gray and white matters compressed and displaced by the mass effect. In the intratumoral measurements, the regions suspected of necrosis, cysts, hemorrhage, vessels, and calcification were excluded from the ROIs whenever possible in order to avoid false lesion estimation. In human studies, DWI and DTI analyses of brain tumors have been performed using ADC or FA ratios that are calculated by dividing the actual (intratumoral or peritumoral) values by the values of the contralateral NAWM (21, 22). In order to compare ADC and FA among different species (dogs and cats), we needed to evaluate whether the actual ADC and FA values could be used or whether the ADC or FA ratio should be used instead. For that purpose, we measured ADC and FA in the NAWM of the hemisphere contralateral to the tumor. When the selection of the five ROIs for the contralateral NAWM, the operator selected the NAWM area that was not affected by mass effect as much as possible without edematous finding on the contralateral hemisphere to the tumoral side. ROI of peritumoral and NAWM were also measured in the area ranging from 7.7 to 17.2 mm^2^.

### Statistical Analysis

Statistical analyses were performed by another investigator (AFI) in a blinded manner using commercial software (Statcel4; OMS, Tokyo, Japan). The Mann-Whitney *U* test was employed to compare the DWI and DTI parameters (i.e., ADC and FA, respectively) between canine and feline meningiomas and between canine and feline NAWM. In all analyses, *p* < 0.05 was considered to be statistically significant.

## Results

Thirteen dogs consisting of seven males (three neutered) and six females (two neutered), and 6 cats consisting of three males (two neutered) and three females (two neutered), fulfilled the inclusion criteria of this study. Signalment and histopathological diagnoses of the cases in this study are summarized in Table 1. The mean ages of the patients with canine and feline meningioma were 11.7 years (range, 8.6–15.3 years) and 14.1 years (range, 11.6–16.0 years), respectively. The breeds of the dogs were three Miniature Dachshunds, two Toy Poodles, and one each of Beagle, Shetland Sheepdog, Labrador Retriever, Flat-coated Retriever, Shiba Inu, Jack Russell Terrier, Welsh Corgi, and mixed breed. The breeds of the cats were five domestic short-haired cats and one Norwegian Forest cat. Clinical signs, tumor location(s), size, findings on routine MRI, and outcome of each patient are provided in Supplementary Material 1. The histopathological subtypes of meningioma consisted of six transitional (mixed), three meningotheliomatous, two anaplastic, and one each of fibrous and papillary in the dogs, and five transitional (mixed) and two psammomatous in the cats. One cat had two different types of meningioma in the cerebrum. In addition, one dog only received a DWI examination and another dog only received a DTI examination. Therefore, we measured 12 lesions in dogs for each of ADC and FA analysis and seven lesions in cats for both ADC and FA analyses.

**Table 1:** Summary of signalment and histopathological type in the cases.

**Case**	**Breed**	**Age (years)**	**Sex**	**Histopathological Type**
Dog 1	Beagle	10	F	Transitional (mixed)
Dog 2	Toy Poodle	10	M	Transitional (mixed)
Dog 3	Toy Poodle	12	MC	Transitional (mixed)
Dog 4	Miniature Dachshund	12	M	Transitional (mixed)
Dog 5	Miniature Dachshund	13	M	Transitional (mixed)
Dog 6	Shetland Sheepdog	13	F	Transitional (mixed)
Dog 7 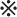	Welsh Corgi	13	M	Meningotheliomatous
Dog 8	Labrador Retriever	12	FS	Meningotheliomatous
Dog 9	Shiba Inu	8	MC	Meningotheliomatous
Dog 10	Mixed Breed	10	F	Anaplastic
Dog 11	Welsh Corgi	15	MC	Anaplastic
Dog 12	Miniature Dachshund	9	F	Papillary
Dog 13 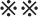	Flat-coated Retriever	9	FS	Fibrous
Cat 1	Domestic Short-haired	11	M	Transitional (mixed)
Cat 2	Domestic Short-haired	12	F	Transitional (mixed)
Cat 3	Domestic Short-haired	16	FS	Transitional (mixed)
Cat 4	Domestic Short-haired	14	MC	Transitional (mixed)
Cat 5	Domestic Short-haired	15	FS	Transitional (mixed)
				Psammomatous
Cat 6	Norwegian Forest Cat	13	MC	Psammomatous

*M, male; F, female; MC, castrated male; FS, spayed female, 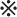
only DTI, 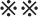
only DWI. More detail data is shown in Supplementary Material 1*.

In each species, the median and range of the actual ADC and FA values in the intratumoral (large ROI and small ROIs), peritumoral, and contralateral NAWM were measured (data is shown in Supplementary Material 2).

### Intratumoral, Peritumoral and NAWM ADC and FA Values

**Figure 3:**
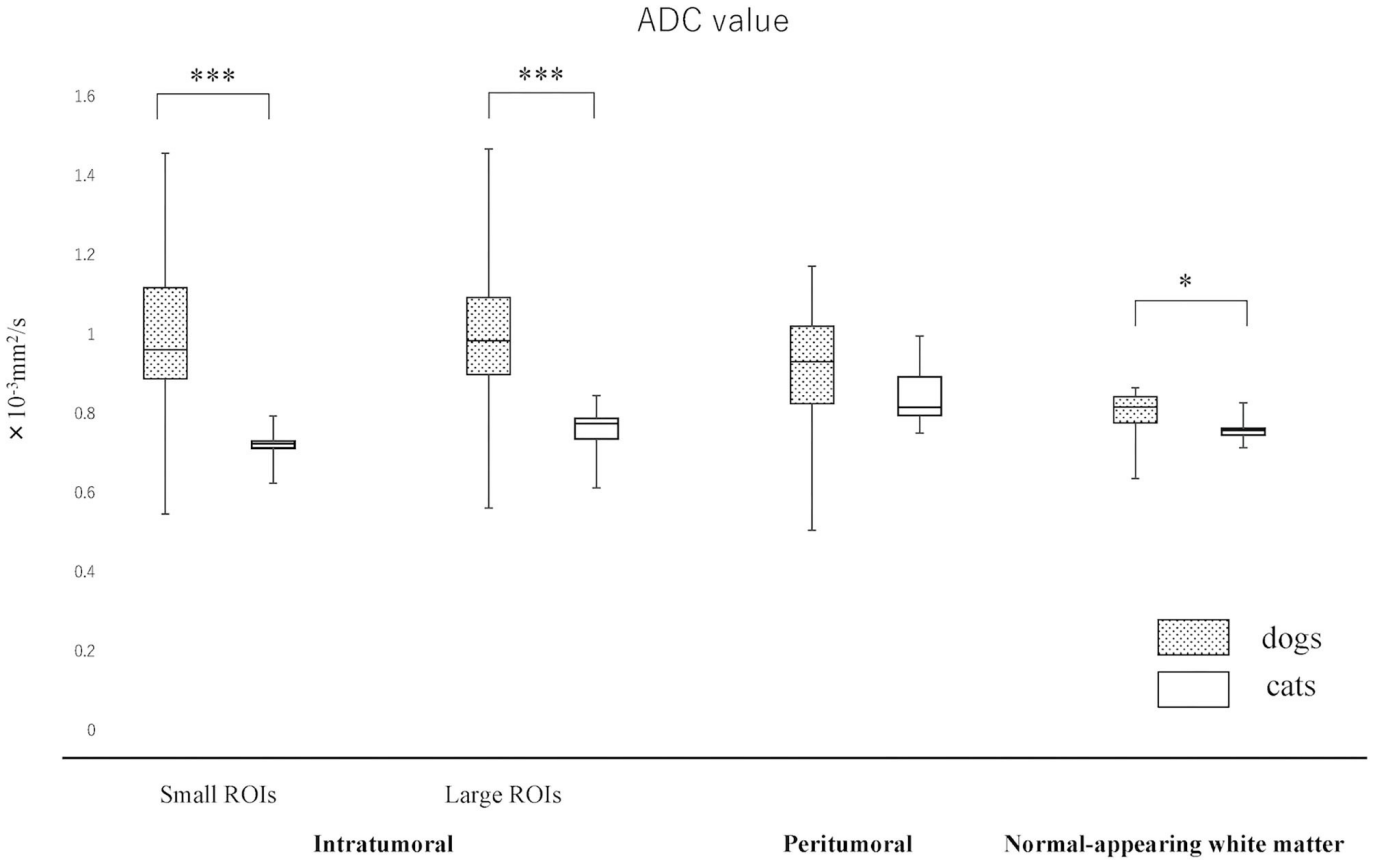
Boxplots comparing canine and feline meningiomas for apparent diffusion coefficient (ADC) values in the intratumoral [small regions of interest (ROIs) and large ROIs] and peritumoral areas, and in the contralateral normal-appearing white matter (as an internal standard). The middle line and upper/lower box edges of each box represent the median and upper/lower quartiles, respectively. Both the end of upper/lower whiskers show the maximum/minimum values. Statistical analyses were performed using the Mann–Whitney *U* test. *p < 0.05, ***p < 0.0001.

**Figure 4:**
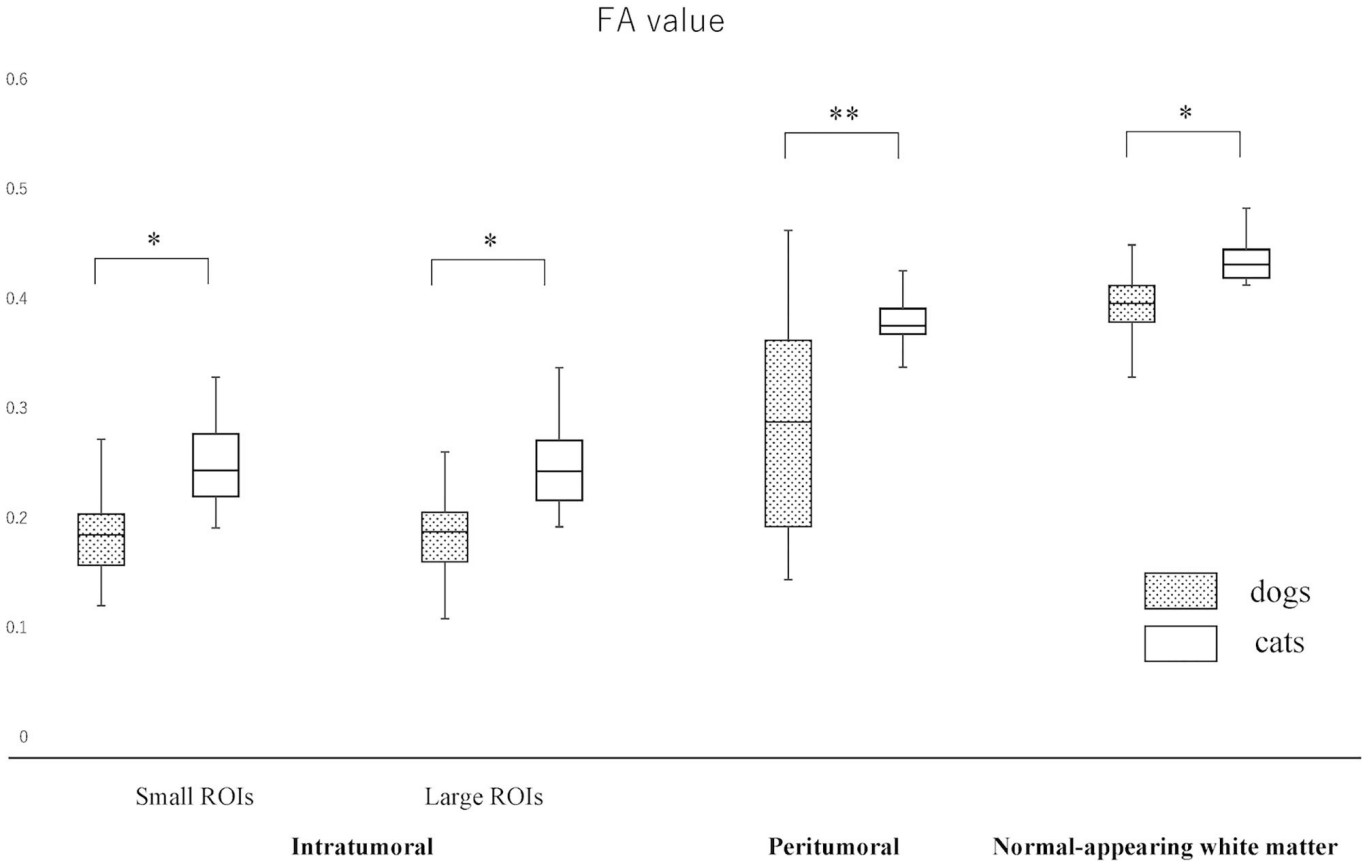
Boxplots comparing canine and feline meningiomas for fractional anisotropy (FA) values in the intratumoral [small regions of interest (ROIs) and large ROIs] and peritumoral areas, and in the contralateral normal-appearing white matter (as an internal standard). The middle line and upper/lower box edges of each box represent the median and upper/lower quartiles, respectively. Both the end of upper/lower whiskers show the maximum/minimum values. Statistical analyses were performed using the Mann–Whitney *U* test. *p < 0.05, **p < 0.005.

The actual ADC and FA values of intratumoral small and large ROIs, peritumoral region, and NAWM are summarized in Figures 3, 4 and Supplementary Material 2. The ADC values (median; range × 10^−3^ mm^2^/s) for intratumoral small ROIs were significantly lower in cats (0.71; 0.64–0.77) than in dogs (0.96; 0.54–1.17) (*p* < 0.0001). Similarly, the ADC values for intratumoral large ROIs were significantly lower in cats (0.77; 0.62–0.86) than in dogs (1.00; 0.57–1.18) (*p* < 0.0001). The FA values (median; range) for intratumoral small ROIs were significantly higher in cats (0.24; 0.20–0.29) than in dogs (0.18; 0.10–0.29) (*p* = 0.01). Similarly, the FA values for intratumoral large ROIs were significantly higher in cats (0.23; 0.19–0.34) than in dogs (0.19; 0.10–0.26) (*p* < 0.02). In the peritumoral region, there was no significant difference in the ADC values (median; range × 10^−3^ mm^2^/s) between dogs (0.95; 0.51–1.16) and cats (0.83; 0.76–0.99) (*p* = 0.08). On the other hand, the FA values (median; range with no unit) for the peritumoral region were significantly higher in cats (0.38; 0.31–0.42) than in dogs (0.29; 0.14–0.44) (*p* < 0.001). In contralateral NAWM, the ADC values (median; range × 10^−3^ mm^2^/s) were significantly higher in dogs (0.82; 0.64–0.87) than in cats (0.76; 0.71–0.83) (*p* = 0.02), and the FA values (median; range) were significantly lower in dogs (0.39; 0.36–0.43) than in cats (0.43; 0.41–0.48) (*p* = 0.01).

### Intratumoral, Peritumoral and NAWM ADC and FA Ratios

**Figure 5:**
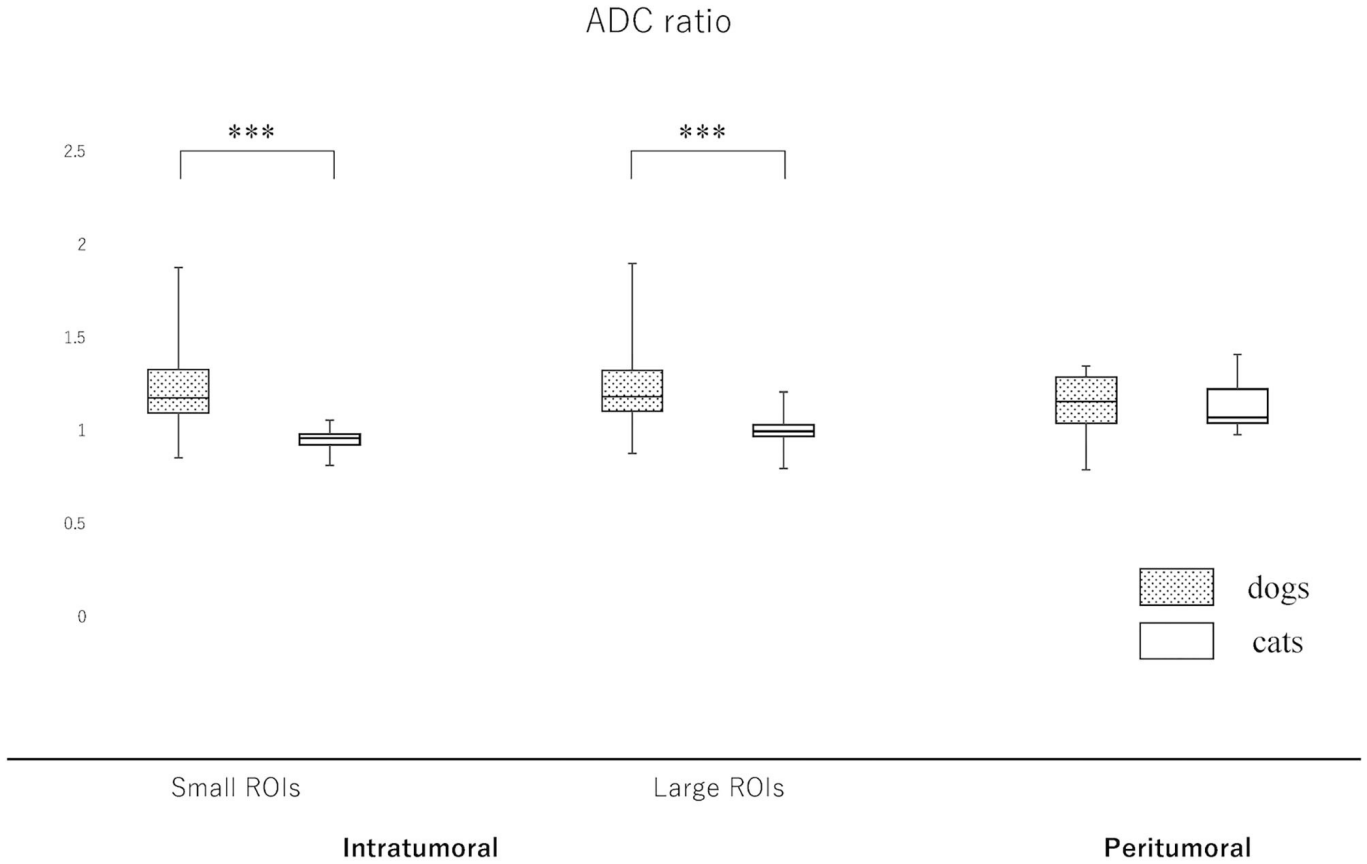
Boxplots comparing canine and feline meningiomas for the apparent diffusion coefficient (ADC) ratio in the intratumoral [small regions of interest (ROIs) and large ROIs] and peritumoral areas. The ADC ratio was calculated by dividing the intratumoral and peritumoral values by the value of the contralateral normal-appearing white matter. The middle line and upper/lower box edges of each box represent the median and upper/lower quartiles, respectively. Both the end of upper/lower whiskers show the maximum/minimum values. Statistical analyses were performed using the Mann–Whitney *U* test. ***p < 0.0001.

**Figure 6:**
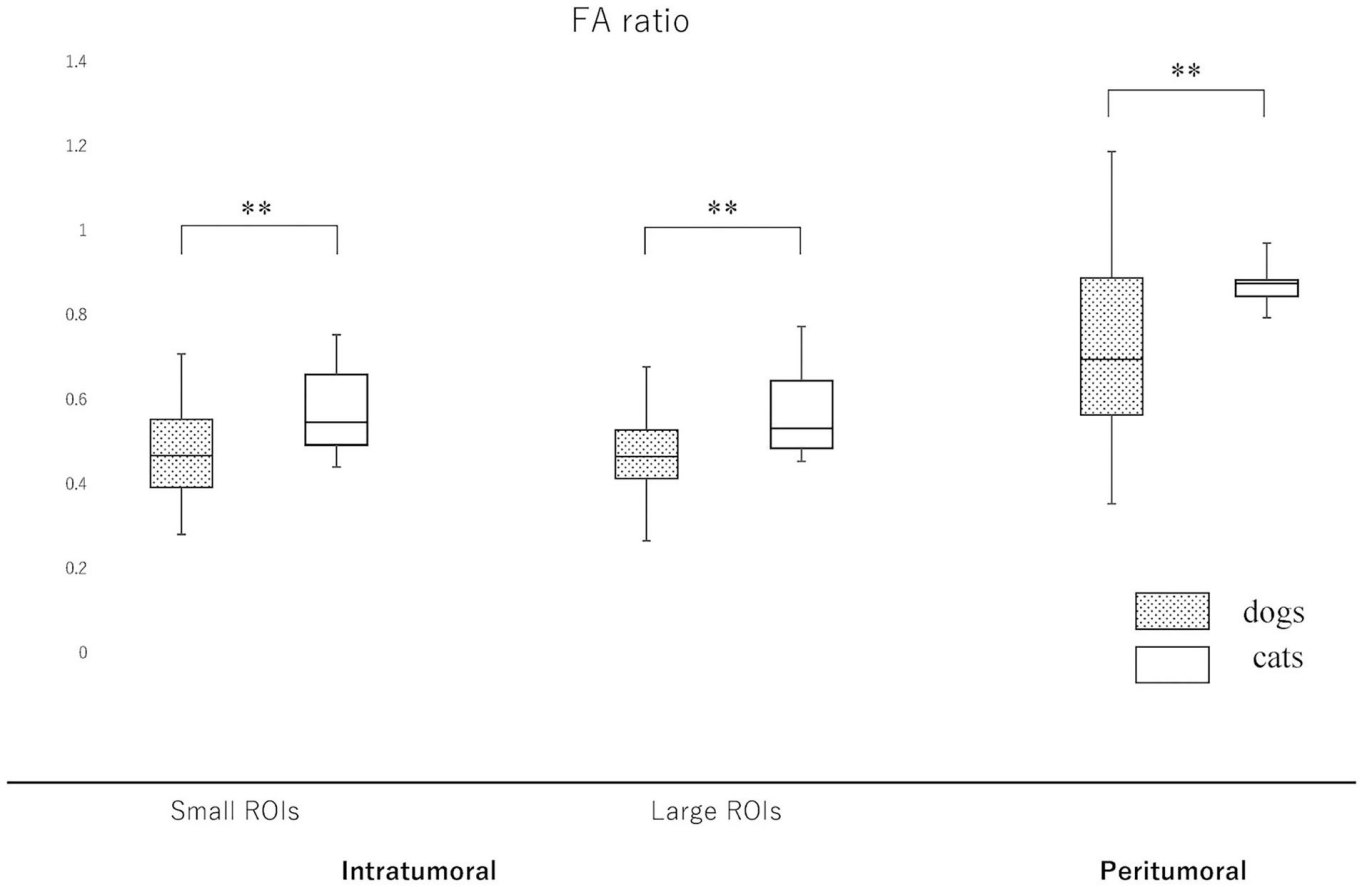
Boxplots comparing canine and feline meningiomas for the fractional anisotropy (FA) ratio in the intratumoral [small regions of interest (ROIs) and large ROIs] and peritumoral areas. The FA ratio was calculated by dividing the intratumoral and peritumoral values by the value of the contralateral normal-appearing white matter. The middle line and upper/lower box edges of each box represent the median and upper/lower quartiles, respectively. Both the end of upper/lower whiskers show the maximum/minimum values. Statistical analyses were performed using the Mann–Whitney *U* test. **p < 0.005.

Since the NAWM values were significantly different between dogs and cats, we judged it difficult to compare dogs and cats using the actual ADC and FA values. Thus, it was considered to be reasonable to use the ADC and FA ratios, that were calculated as dividing actual ADC or FA values of intratumoral or peritumoral ROIs by those of NAWM, for comparing between dogs and cats. So that the ADC and FA ratios of intratumoral small and large ROIs and the peritumoral ROIs were calculated in each case and reanalyzed to compare canine and feline meningiomas (Figures 5, 6, Supplementary Material 2). The ADC ratio for intratumoral small ROIs was significantly lower in cats (0.94; 0.84–1.04) than in dogs (1.20; 0.84–1.53) (*p* < 0.0001). Similarly, the ADC ratio for intratumoral large ROIs was significantly lower in cats (1.01; 0.81–1.14) than in dogs (1.21; 0.89–1.51) (*p* < 0.0001). Conversely, the FA ratio for intratumoral small ROIs was significantly higher in cats (0.54; 0.47–0.75) than in dogs (0.45; 0.25–0.77) (*p* = 0.002). Similarly, the FA ratio for intratumoral large ROIs was significantly higher in cats (0.56; 0.45–0.77) than in dogs (0.49; 0.27–0.67) (*p* < 0.001). In the peritumoral region, there was no difference in the ADC ratio between dogs (1.15; 0.80–1.34) and cats (1.10; 0.99–1.27) (*p* = 0.43). However, there was a significant difference between the FA ratio for peritumoral region between dogs (0.70; 0.34–1.03) and cats (0.85; 0.72–1.01) (*p* = 0.002).

## Discussion

In this study, there was a significant difference in the ADC and FA values of dogs and cats in the NAWM on the contralateral side to the lesion. Therefore, when comparing dogs and cats, it is recommended to use a relative value, i.e., ADC and FA ratios, obtained by dividing the value for the tumor with the value for NAWM in each species instead of using the actual value.

In agreement with our hypothesis, we found that feline meningiomas had lower ADC values and higher FA values than canine meningiomas. In general, feline meningiomas are less aggressive and more likely to undergo successful total resection compared to canine meningiomas (23). The consistency of meningiomas is one of the most important factors for achieving total resection with minimizing neurological deficits (24). Therefore, the prediction in consistency of meningiomas based on imaging findings may influence the surgical strategy and requirement for additional surgical equipment such as an ultrasonic surgical aspirator and surgical microscope (24). The tumor cells of meningioma are characterized by interdigitations connected with junctional complexes and extracellular cisterns, and it is considered that meningiomas with high cellularity have strong cell adhesion (25). It has been suggested that the ADC is inversely correlated with tumor cellularity and the amount of fibrous tissue within heterogeneous tumors (11, 26, 27). Therefore, if the number of cells and/or contained fibrous tissues are high, the ADC value will be low (28). Conversely, in human medicine, the FA value is statistically significant with regard to the consistency of meningiomas and associated with: (1) a very soft tumor for FA <0.1; (2) a soft tumor when FA is between 0.1 and 0.2; (3) a hard tumor when FA is between 0.2 and 0.3; and (4) a very hard tumor for FA > 0.3 (10). In the present study, significantly lower ADC and higher FA values and ratios of meningiomas were observed in cats compared to dogs. In particular, the FA value of feline meningiomas showed a range of 0.19–0.34 (median 0.25), and these are classified as hard tumors or very hard tumors (classification 3 or 4) in the classification system of Romani et al. (10). While most of those in dogs (median 0.18) were classified as soft tumors. This suggested that the meningiomas of cats would have higher cell density or fibrous tissue and be harder than those of dogs. This result in MRI is consistent with previous histopathological reports that feline meningiomas are often fibrous and hard (8, 29). In the present study, we indicated that advanced MRI sequences (DWI and DTI) with ADC and FA value measurements may be required to predict the consistency of meningiomas accurately.

DWI can distinguish vasogenic edema from cytotoxic edema and gliosis related to meningiomas in humans (30). In peritumoral edema of metastases, fiber tract disruption similar to diffuse axonal injury has been reported (31). In contrast to metastases, fiber tracts are relatively well-preserved in the peritumoral tissue of meningiomas in humans; the FA ratio in peritumoral tissue was 0.32 for metastases and 0.45 for meningiomas (32, 33). In studies of the spinal cord, at compression sites without spinal cord injury such as edema and demyelination, the ADC values decrease and the FA values increase, whereas at compression sites with spinal cord injury, the ADC values increase and the FA values decrease (34, 35). From these facts, we speculated that FA will be increased and ADC will be decreased in the peritumoral regions of feline meningiomas. As a result of the present study, FA ratio of the peritumoral region in cats was significantly higher than that in dogs, although there was no significant difference in the ADC ratio. This may suggest that the peritumoral brain tissues in cats were more well-preserved or more densely compressed white matter fibers exist around the tumor. On the other hand, the actual ADC value of feline NAWM was lower than that of dogs, and actual FA value was higher. The loss of white matter myelin has been reported in older dogs (36), and the cases of the present study were also older dogs. Therefore, although the effect of aging cannot be ruled out, we speculate that the feline brain may originally have denser fibers and be slightly firmer and/or may have less water content than the canine brain. This may be related to the observation that the peritumoral brain remains compressed even after meningioma removal in feline cases.

The present study has several limitations. The principal limitation of this study is its relatively small number of patients. Therefore, we were not able to carry out statistical analysis comparing each meningioma subtype, because there was a small number of each subtype, except for the transitional (mixed) type. Second, since this study was retrospective in nature, we were unable to perform histological quantitative analysis regarding cell density and fibrous tissue content in the tumors. We hope that imaging analysis associated with histologic classification and quantitative histological analysis can be performed by collecting more cases in the future. Third, all ROIs were drawn manually and their size might be a source of bias when comparing different studies. Actually, there is no recommendation about the optimal size of ROI to use. Large ROIs have a high probability of including undetected microcystic or necrotic areas. Conversely, the mean ADC and FA values of small ROIs might vary with small changes in ROI positioning, even in NAWM, due to the relatively high standard deviation of ADC and FA values (37). In the present study, because both methods showed similar results; therefore, there is a possibility that reliable numerical values will be obtained for meningioma regardless of the method. However, in order to confirm the accuracy of this result, it is necessary to increase the number of cases.

If it is possible to predict whether a tumor is easy to remove before surgery, it will provide valuable information affecting risk assessment, patient management, and workflow optimization. Although many factors such as tumoral size, location, histological subtype, malignancy, clearness of tumoral margin or infiltration, use of neurosurgical tools, and skill of surgeon contribute to the easiness of removal, the tumor solidity is considered to be one of the important factors. Finally, we propose that presurgical ADC and FA measurements could predict a tumor's consistency and may provide useful information for considering operation techniques.

## Data Availability Statement

All datasets generated for this study are included in the article/Supplementary Material. Further inquiries can be directed to the corresponding author.

## Ethics Statement

Ethical review and approval was not required for the animal study because this study is the retrospective imaging analysis study in nature, the ethical consent for animal use was not required. Nevertheless, all owners of the dogs and cats included in this study had agreed to the use of their data for academic purposes, and had previously signed a consent form at the first presentation to the teaching hospital. Written informed consent was obtained from the owners for the participation of their animals in this study.

## Author Contributions

MW and DH: conception and design. DH, YH, YY, and RA: clinical consultation and acquisition of data. MW, YY, and AF-I: data and statistical analysis. MW: drafting the first manuscript. MW, DH, YY and MF: editing and revising manuscript. All authors contributed to the article and approved the submitted version.

## Conflict of Interest

MW is employed by the company ORM Co, Ltd., which provides veterinary diagnostic imaging advisory services. The remaining authors declare that the research was conducted in the absence of any commercial or financial relationships that could be construed as a potential conflict of interest.
