# Supplementary material for: Comparison of Canine and Feline Meningiomas Using the Apparent Diffusion Coefficient and Fractional Anisotropy
Source: Front Vet Sci. 2021 Jan 11;7:614026. doi: 10.3389/fvets.2020.614026 (PMC7829344; doi:10.3389/fvets.2020.614026)
Supplement: Supplementary file 1 [file Data_Sheet_1.PDF]

Supplementary Material 1

Summary of canine and feline cases with meningioma in the study

Case#	Breed	Age (Y)	Sex	Clinical signs	Location	M vol (cm3)	IC vol (cm3)	Occup %	T2W	T1W	CE	C/N	Peri Edm	Mass Effect	Hist	Outcome
Dog 1	Beagle	10	F	Sz, L:PR	R: Flx-Frn	6.7	72.7	9.2	± ~ +	±	+	+	++	++	TR	RO:1y, ST: >1.5y (LOF)
Dog 2	T. P	10	M	Sz, L:Circl, R:PR	L: Frn	3.4	56.4	6.0	++	+	++	+	++	++	TR	ST: >2.5y (LOF)
Dog 3	T. P	12	MC	Sz	L: Flx-Frn	0.2	63.4	0.2	±	±	+	–	–	–	TR	ST: >3y (LOF)
Dog 4	M. D	13	M	Sz, Atx, L: MNR, PR	R: Frn	3.3	65.0	5.1	±	± ~ +	+	+	+	++	TR	ST: 3y8m (DBO)
Dog 5	M. D	13	M	Sz, L:MNR, PR	R: Frn	2.7	64.8	4.2	± ~ +	– ~ ±	++	+	+++	++	TR	RO:1.5y, ST: >2y (LOF)
Dog 6	S. S	13	F	Sz, R:Circl, L:PR	R: Flx-Frn	3.0	63.5	4.7	± ~ +	+	+	–	++	++	TR	ST: 1d
Dog 7	W. C	13	M	Sz, Atx	R: Olf-Frn	1.0	75.3	1.3	±	±	+	+	++	+	MNT	ST: 2y4m
Dog 8	L. Ret	12	FS	DP, Sz, Atx, L: Circl, R:PR	L: Flx-Frn	3.3	103.0	3.2	± ~ +	±	+	+	+	+++	MNT	ST: 1y3m (DBO)
Dog 9	Shiba	8	MC	Sz	R: Frn	0.7	53.2	1.3	±	± ~ +	+	–	+	+	MNT	RT:1y5m, ST: >2y (LOF)
Dog 10	Mix	10	F	CD, Sz, L:PR	R: Olf-Frn	1.2	80.3	1.5	+	–	+	+	++	++	ANA	ST: >1y (LOF)
Dog 11	W.C	15	MC	CD, Sz, Coma	R: Frn	4.8	83.0	5.8	– ~ +	– ~ +	+	+	++	+++ TTH, FMH	ANA	ST: 0d (MRI-EU)
Dog 12	M. D	9	F	CD, HP, Sz, L:Circl, LR:PR	LR: Flx-Frn-Par	3.9	50.4	7.7	±	±	++	+	+	++	PAP	RO: 11m, ST: 1y4m
Dog 13	F. Ret	9	FS	Sz, R:PR	L: Olf-Frn	3.0	91.3	3.3	± ~ +	–	+	–	++	+	FIB	ST: 2y9m (DBO)
Cat 1	DSH	11	M	Sz, L:Circl, R:MNR,PR	R: Par-Occ	4.1	33.4	12.3	± ~ +	–	++	+	–	+++ TTH,	TR	ST: 1d (EU)

														FMH		
Cat 2	DSH	12	F	Sz, L:Circl, R:MNR,PR	LR: Flx-Frn-Par	2.0	30.0	6.7	± ~ +	–	+	–	+	++	TR	ST: 0d (EU)
Cat 3	DSH	16	FS	CD, DP	LR: Flx-Par-Occ	3.0	29.0	10.3	± ~ +	–	+	–	–	++ TTH	TR	ST: 6m (LOF)
Cat 4	DSH	14	MC	DP, Atx, R:PR	L: Frn	3.2	35.3	9.2	± ~ +	–	+	+	+	+++ TTH, FMH	TR	ST: >3y (LOF)
Cat 5	DSH	15	FS	DP, Atx, L:Circl, R:PR, LR:MNR	L: Frn	1.2	28.7	4.2	–	–	+	–	+	++ TTH	TR	ST: >2y (LOF)
					L: Par	1.2	28.7	4.2	+	±	+	+	–		PSM	
Cat 6	NFC	13	MC	Sz, DP, Atx, R:MNR, LR:PR	LR: Flx-Par-Occ	2.7	32.7	8.3	– ~ ±	– ~ ±	+	+	–	+++ TTH, FMH	PSM	ST: 8m (DBU)

Abbreviations and Indications:

Title: M vol, mass volume; IC vol, intracranial volume; Occup %, occupying percentage (= mass volume/intracranial volume x100); T2W, signal intensity of T2-weighted images of the mass; T1W, signal intensity of T1-weighted images of the mass; CE, contrast enhancement; C/N, the presence of cystic or necrotic area within the mass; Peri Edm, the presence and degree of peritumoral edema; Mass Effect, the presence and degree of the mass effect; Hist, histological type of meningioma.

Breed: T. P, Toy Poodle; M. D, Miniature Dachshund; S. S, Shetland Sheepdog; W. C, Welsh Corgi; L. Ret, Labrador Retriever; Shiba, Shiba Inu; F. Ret, Flat-coated Retriever; DSH, domestic short-haired; NFC, Norwegian Forest Cat.

Sex: M, male; MC, castrated male; F, female; FS, spayed female.

Clinical signs: L, left; R, right; LR, left and right; CD, cognitive dysfunction; DP, depression (obtunded); HP, head pressing; Atx, ataxia; Circl, circling; Sz, seizures; MNR, decreased or loss of menace response; PR, decreased or loss of postural reactions.

Location: L, left; R, right; LR, extended to the left and right; Olf, olfactory; Flx, falx; Frn, frontal; Par, parietal; Tem, temporal; Occ, occipital.

Mass and intracranial volume: volumetry using CE-T1W images by ROI method.

T2W (signal intensity): – –, hypointensity as bone; –, hypointensity; ±, isointensity to gray matter; +, hyperintensity; ++, hyperintensity as CSF.

T1W (signal intensity): – –, hypointensity as CSF; –, hypointensity; ±, isointensity to gray matter; +, hyperintensity; ++, hyperintensity as fat.

CE (contrast enhancement): –, absent; +, moderate enhancement; ++, strong enhancement.

C/N (cystic or necrotic area): –, absent; +, present.

Peritumoral edema: –, absent to unclear; +, mild; ++, moderate; +++, severe.

Mass effect: –, absent to unclear; +, mild; ++, moderate; +++, severe; TTH, transtentorial herniation; FMH, foramen magnum herniation.

Histological type: TR, transitional; MNT, meningotheliomatous; ANA, anaplastic; PAP, papillary; FIB, fibrous; PSM, psammomatous.

Outcome: ST, survival time after surgery; RO, re-operation; LOF, lost to follow up; EU, euthanized; DBO, dead by other cause; DBU, dead by unknown cause; RT, radiation; d, day; m, month; y, year.
