# Supplementary material for: Comparison of Canine and Feline Meningiomas Using the Apparent Diffusion Coefficient and Fractional Anisotropy
Source: Front Vet Sci. 2021 Jan 11;7:614026. doi: 10.3389/fvets.2020.614026 (PMC7829344; doi:10.3389/fvets.2020.614026)
Supplement: Supplementary file 2 [file Data_Sheet_2.PDF]

Supplementary Material 2:

1. Data sheet of ADC measurements in canine meningioma. Median (min-max)

Case#	ADC (x $10^{-3}mm^2/s$)				ADC ratio (/NAWM)		
	Intratumoral		Peritumoral	NAMW	Intratumoral		Peritumoral
	Large ROI	Small ROI			Large ROI	Small ROI	
Dog 1	1.18(1.17-1.25)	1.19(1.19-1.39)	0.83(0.81-0.84)	0.80(0.71-0.83)	1.51(1.50-1.60)	1.53(1.40-1.78)	1.07(1.04-1.08)
Dog 2	1.004(0.99-1.1)	1.14(0.94-1.26)	0.82(0.74-0.89)	0.84(0.79-0.87)	1.26(1.20-1.33)	1.38(1.1.3-1.52)	0.99(0.90-1.07)
Dog 3	1.00(0.80-1.05)	0.83(0.79-1.08)	0.98(0.91-1.11)	0.86(0.81-0.88)	1.18(0.93-1.24)	0.98(0.93-1.27)	1.15(1.07-1.31)
Dog 4	1.14(1.10-1.49)	1.18(1.03-1.33)	1.16(1.13-1.25)	0.90(0.74-0.94)	1.31(1.26-1.77)	1.35(1.19-1.53)	1.34(1.30-1.44)
Dog 5	0.95(0.86-0.96)	0.93(0.89-1.02)	0.97(0.88-1.09)	0.82(0.80-0.92)	1.12(1.02-1.14)	1.10(0.99-1.18)	1,15(1.02-1.29)
Dog 6	0.89(0.86-0.92)	0.88(0.83-0.91)	1.10(1.08-1.17)	0.83(0.78-0.85)	1.07(1.00-1.10)	1.08(1.05-1.12)	1.34(1.31-1.43)
Dog 7	–	–	–	–	–	–	–
Dog 8	1.62(0.90-1.67)	1.45(1.34-1.61)	0.95(0.81-1.03)	0.82(0.69-0.83)	2.09(1.17-2.05)	1.87(1.74-2.08)	1.23(1.05-1.33)
Dog 9	1.06(1.02-1.11)	1.05(0.97-1.28)	1.14(0.98-1.23)	0.86(0.83-0.93)	1.23(1.18-1.28)	1.23(1.13-1.48)	1.32(1.13-1.43)
Dog 10	1.00(0.95-1.11)	1.02(0.97-1.06)	0.79(0.67-0.87)	0.78(0.77-0.80)	1.27(1.21-1.41)	1.30(1.11-1.35)	1.60(1.41-1.75)
Dog 11	0.57(0.55-0.58)	0.54(0.53-0.60)	0.51(0.41-0.58)	0.64(0.61-0.69)	0.89(0.85-0.90)	0.84(0.83-0.93)	0.80(0.65-0.91)
Dog 12	0.81(0.75-0.91)	0.84(0.67-0.87)	0.92(0.80-0.93)	0.74(0.64-0.77)	1.13(1.04-1.25)	1.16(0.93-1.21)	1.28(1.10-1.54)
Dog 13	0.88(0.87-1.01)	0.91(0.81-0.93)	0.90(0.80-0.91)	0.81(0.77-0.91)	1.08(1.07-1.23)	1.12(1.07-1.22)	1.10(0.98-1.11)
Med	1.00	0.98	0.94	0.82	1.21	1.20	1.23
Min	0.57	0.54	0.51	0.64	0.89	0.84	0.80
Max	1.62	1.45	1.16	0.90	2.09	1.87	1.60
IQR	0.89-1.08	0.87-1.15	0.83-1.01	0.80-0.85	1.11-1.28	1.10-1.36	1.09-1.33

2. Data sheet of FA measurements in canine meningioma. Median (min-max)

Case#	FA				FA ratio (/NAWM)		
	Intratumoral		Peritumoral	NAMW	Intratumoral		Peritumoral
	Large ROI	Small ROI			Large ROI	Small ROI	
Dog 1	0.17(0.15-0.23)	0.22(0.20-0.30)	0.44(0.22-0.46)	0.43(0.42-0.46)	0.40(0.35-0.54)	0.51(0.46-0.70)	1.03(0.50-1.16)
Dog 2	0.21(0,20-0.22)	0.19(0.16-0.24)	0.36(0.31-0.41)	0.40(0.37-0.44)	0.52(0.49-0.54)	0.40(0.39-0.54)	0.84(0.78-1.01)
Dog 3	0.16(0.16-0.19)	0.18(0.17-0.20)	0.19(0.15-0.20)	0.37(0.28-0.40)	0.48(0.40-0.50)	0.48(0.43-0.52)	0.49(0.38-0.51)
Dog 4	0.20(0.20-0.21)	0.21(0.19-0.25)	0.30(0.17-0.41)	0.36(0.34-0.42)	0.56(0.55-0.58)	0.57(0.52-0.69)	0.83(0.46-1.13)
Dog 5	0.20(0.17-0.21)	0.15(0.13-0.22)	0.29(0.25-0.29)	0.41(0.36-0.48)	0.49(0.40-0.51)	0.38(0.31-0.56)	0.70(0.62-0.74)
Dog 6	0.26(0.25-0.27)	0.30(0.22-0.31)	0.29(0.21-0.33)	0.35(0.32-0.48)	0.67(0.64-0.74)	0.77(0.56-0.81)	0.74(0.54-0.86))
Dog 7	0.21(0.18-0.25)	0.20(0.16-0.22)	0.18(0.15-0.29)	0.31(0.30-0.37)	0.63(0.54-0.75)	0.60(0.47-0.75)	0.55(0.46-0.87)
Dog 8	0.10(0.10-0.13)	0.10(0.10-0,16)	0.14(0.11-0.17)	0.41(0.37-0.45)	0.26(0.24-0.31)	0.25(0.23-0.40)	0.34(0.27-0.44)
Dog 9	0.16(0.14-0.19)	0.15(0.10-0.18)	0.15(0.13-0.17)	0.34(0.32-0.40)	0.45(0.40-0.55)	0.42(0.30-0.51)	0.42(0.38-0.49)
Dog 10	0.20(0.19-0.21)	0.20(0.16-0.25)	0.43(0.36-0.56)	0.39(0.34-0.44)	0.52(0.49-0.52)	0.52(0.40-0.64)	0.57(0.23-0.88)
Dog 11	0.16(0.11-0.17)	0.17(0.11-0.21)	0.37(0.32-0.51)	0.45(0.36-0.56)	0.36(0.24-0.39)	0.40(0.26-0.47)	0.85(0.72-1.17)
Dog 12	0.12(0.11-0.13)	0.12(0.11-0.14)	0.31(0.27-0.34)	0.45(0.37-0.57)	0.27(0.26-0.33)	0.26(0.23-0.38)	0.70(0.61-0.76)
Dog 13	–	–	–	–	–	–	–
Med	0.19	0.19	0.30	0.40	0.49	0.45	0.70
Min	0.10	0.10	0.14	0.31	0.26	0.25	0.34
Max	0.26	0.30	0.44	0.45	0.67	0.77	1.03
IQR	0.16-0.20	0.15-0.20	0.19-0.36	0.36-0.42	0.39-0.53	0.40-0.53	0.54-0.83

3. Data sheet of ADC measurements in feline meningioma. Median (min-max)

Case#	ADC (x 10^{-3}mm^2/s)				ADC ratio (/NAWM)		
	Intratumoral		Peritumoral	NAMW	Intratumoral		Peritumoral
	Large ROI	Small ROI			Large ROI	Small ROI	
Cat 1	0.79(0.71-0.84)	0.73(0.68-0.84)	0.82(0.65-0.90)	0.75(0.69-0.76)	1.06(0.95-1.13)	0.92(0.90-1.13)	1.10(0.87-1.13)
Cat 2	0.62(0.57-0.63)	0.64(0.60-0.68)	0.76(0.74-0.86)	0.75(0.74-0.80)	0.81(0.74-0.83)	0.84(0.75-0.89)	0.99(0.82-1.13)
Cat 3	0.78(0.76-0.83)	0.72(0.71-0.83)	0.88(0.80-0.97)	0.84(0.80-0.86)	0.94(0.91-1.00)	0.87(0.85-1.00)	1.06(0.96-1.17)
Cat 4	0.71(0.70-0.72)	0.71(0.69-0.77)	0.83(0.76-0.86)	0.74(0.59-0.80)	1.01(0.98-1.01)	0.98(0.96-1.08)	1.16(1.00-1.17)
Cat 5	0.76(0.74-0.85)	0.71(0.68-0.83)	0.97(0.83-1.21)	0.76(0.70-0.83)	1.00(0.98-1.12)	0.94(0.89-1.09)	1.27(1.06-1.55)
Cat 6	0.77(0.72-0.80)	0.72(0.68-0.78)	0.80(0.72-0.84)	0.74(0.68-0.84)	1.01(0.94-1.02)	0.94(0.89-1.01)	1.05(0.95-1,10)
Cat 7	0.86(0.80-0.89)	0.78(0.74-0.86)	0.91(0.86-0.97)	0.75(0.71-0.80)	1.14(1.05-1.16)	1.04(0.99-1.15)	1.21(1.15-1.30)
Med	0.77	0.72	0.83	0.75	1.01	0.94	1.10
Min	0.62	0.64	0.76	0.74	0.81	0.84	0.99
Max	0.86	0.78	0.97	0.84	1.14	1.04	1.27
IQR	0.74-0.77	0.71-0.73	0.81-0.90	0.75-0.76	0.97-1.04	0.90-0.96	1.06-1.19

4. Data sheet of FA measurements in feline meningioma. Median (min-max)

Case#	FA				FA ratio (/NAWM)		
	Intratumoral		Peritumoral	NAMW	Intratumoral		Peritumoral
	Large ROI	Small ROI			Large ROI	Small ROI	
Cat 1	0.27(0.24-0.28)	0.29(0.26-0.36)	0.42(0.34-0.45)	0.42(0.32-0.57)	0.64(0.59-0.72)	0.70(0.63-0.73)	1.01(0.83-1.10)
Cat 2	0.22(0.19-0.24)	0.23(0.16-0.34)	0.35(0.27-0.48)	0.39(0.28-0.54)	0.55(0.47-0.58)	0.54(0.40-0.83)	0.85(0.74-1.16)
Cat 3	0.19(0.18-0.22)	0.20(0.16-0.21)	0.38(0.32-0.43)	0.42(0.36-0.50)	0.45(0.41-0.52)	0.48(0.38-0,50)	0.91(0.76-1.00)
Cat 4	0.21(0.19-0.25)	0.24(0.21-0.24)	0.40(0.33-0.41)	0.45(0.40-0.55)	0.45(0.41-0.56)	0.53(0.46-0.61)	0.87(0.72-0.91)
Cat 5	0.28(0.26-0.29)	0.24(0.21-0.36)	0.34(0.31-0.39)	0.45(0.34-0.51)	0.65(0.62-0.69)	0.56(0.49-0.85)	0.80(0.73-0.92)
Cat 6	0.34(0.31-0.36)	0.33(0.29-0.38)	0.31(0.23-0.60)	0.42(0.38-0.44)	0.77(0.73-0.83)	0.75(0.67-0.86)	0.72(0.53-1.37)
Cat 7	0.23(0.22-0.27)	0.23(0.15-0.28)	0.41(0.38-0.49)	0.54(0.28-0.58)	0.47(0.46-0.55)	0.47(0.31-0.58)	0.85(0.78-1.01)
Med	0.23	0.24	0.38	0.42	0.55	0.54	0.85
Min	0.19	0.20	0.31	0.39	0.45	0.47	0.72
Max	0.34	0.33	0.42	0.54	0.77	0.75	1.01
IQR	0.22-0.28	0.23-0.27	0.35-0.41	0.42-0.45	0.46-0.65	0.51-0.63	0.83-0.89
